# Readability and Health Literacy Scores for ChatGPT-Generated Dermatology Public Education Materials: Cross-Sectional Analysis of Sunscreen and Melanoma Questions

**DOI:** 10.2196/50163

**Published:** 2024-03-06

**Authors:** Katie Roster, Rebecca B Kann, Banu Farabi, Christian Gronbeck, Nicholas Brownstone, Shari R Lipner

**Affiliations:** 1 New York Medical College New York, NY United States; 2 Dermatology Department NYC Health + Hospital/Metropolitan New York, NY United States; 3 Department of Dermatology University of Connecticut HealthCenter Framington, CT United States; 4 Department of Dermatology Temple University Hospital Philadelphia, PA United States; 5 Department of Dermatology Weill Cornell Medicine New York, NY United States

**Keywords:** ChatGPT, artificial intelligence, AI, LLM, LLMs, large language model, language model, language models, generative, NLP, natural language processing, health disparities, health literacy, readability, disparities, disparity, dermatology, health information, comprehensible, comprehensibility, understandability, patient education, public education, health education, online information

## Introduction

A study of 402 randomly selected Medicaid enrollees reported an average of a 5th-grade reading level, which is lower than the average 8th-grade level of US adults [1,2]. Therefore, the American Medical Association (AMA) recommends developing health materials at a 6th-grade reading level or lower [3]. However, a 2018 systematic review of 7891 health websites reported that educational health materials are often at 10th- to 15th-grade reading levels [4].

In a study evaluating ChatGPT-generated materials for 14 dermatological diseases, content was at a 10th-grade reading level [5]. We hypothesized that ChatGPT could be prompted to generate rewritten health materials at a lower grade level and in line with AMA recommendations. The readability of ChatGPT-generated dermatology information and public educational resources on the American Academy of Dermatology Association’s (AAD) website was assessed and determined whether strategic prompting would enhance the material’s readability.

## Methods

We inputted the AAD website’s sunscreen and melanoma FAQs individually into ChatGPT, then compiled corresponding outputs, with the supplemental prompts: “I don’t understand, please clarify” and “I still don’t understand, please clarify.” We used well-established readability and health literacy assessment tools and a single web-based readability calculator to calculate 7 different scores [6,7], and computed an “average readability” score with these grade level outputs. A 2-sample *t* test was used for comparisons (*P*<.05). To determine information accuracy before and after prompting, 3 dermatology residents blindly evaluated the education materials using a numerical scale: 1 (not accurate), 2 (somewhat accurate), and 3 (accurate).

## Results

The AAD’s sunscreen FAQs and melanoma FAQs had Flesch Reading Ease scores of 60.9 (standard/average) and 56.2 (fairly difficult), respectively. The initial ChatGPT output had readability scores of 60.5 (standard/average) and 46.5 (difficult) for sunscreen and melanoma questions, respectively. Subsequent prompting resulted in readability levels of 69.4 (standard/average) and 80.2 (easy) for sunscreen questions and 58.9 (fairly difficult) and 59.3 (fairly difficult) for melanoma questions (Table 1).

**Table 1:** Readability and health literacy measures of American Academy of Dermatology Association (AAD) text, ChatGPT output, ChatGPT output with 1 prompt, and ChatGPT output with 2 prompts.

		AAD	ChatGPT	ChatGPT with 1 prompt	ChatGPT with 2 prompts
**Sunscreen FAQs**					
	Flesch Reading Ease score	60.9 (standard/average)	60.5 (standard/average)	69.4 (standard/average)	80.2 (easy)
	Gunning Fog	11.1 (hard)	11.7 (hard)	8.0 (fairly easy)	6.2 (fairly easy)
	Flesch-Kincaid Grade Level	8.9 (9th grade)	9.1 (9th grade)	5.6 (6th grade)	3.8 (4th grade)
	Coleman-Liau Index	10.0 (10th grade)	10.0 (10th grade)	10.0 (10th grade)	8.0 (8th grade)
	SMOG^a^ Index	8.2 (8th grade)	8.6 (9th grade)	6.0 (6th grade)	4.9 (5th grade)
	Automated Readability Index	9.4 (9th grade)	9.4 (9th grade)	4.6 (5th grade)	2.5 (3rd grade)
	Linsear Write Formula	9.3 (9th grade)	10.8 (11th grade)	4.0 (4th grade)	2.8 (3rd grade)
	Average readability^b^	9.2 (9th grade)	9.6 (10th grade)	6.0 (6th grade)	4.4 (4th grade)
**Melanoma FAQs**					
	Flesch Reading Ease score	56.2 (fairly difficult)	46.5 (difficult)	58.9 (fairly difficult)	59.3 (fairly difficult)
	Gunning Fog	12.5 (hard to read)	13.7 (hard to read)	11.0 (hard to read)	10.9 (hard to read)
	Flesch-Kincaid Grade Level	9.5 (10th grade)	10.5 (11th grade)	8.0 (8th grade)	7.9 (8th grade)
	Coleman-Liau Index	9.0 (9th grade)	12.0 (12th grade)	10.0 (10th grade)	8.0 (8th grade)
	SMOG Index	9.4 (9th grade)	10.1 (10th grade)	8.3 (8th grade)	8.2 (8th grade)
	Automated Readability Index	8.4 (8th grade)	9.7 (10th grade)	6.9 (7th grade)	6.3 (6th grade)
	Linsear Write Formula	10.8 (11th grade)	9.5 (10th grade)	7.0 (7th grade)	6.8 (7th grade)
	Average readability	9.4 (9th grade)	10.4 (10th grade)	8.0 (8th grade)	7.4 (7th grade)
Accuracy score^c^, mean (SD)		2.82 (0.25)	2.89 (0.19)	2.63 (0.41)	2.62 (0.37)

^a^SMOG: Simple Measure of Gobbledygook.

^b^The average readability score was computed by averaging the tests with grade levels as outputs: Flesch-Kincaid Grade Level, Coleman-Liau Index, SMOG Index, Automated Readability Index, and Linsear Write Formula.

^c^The accuracy score represents the mean score of 3 dermatology residents who assessed the educational materials using a numeric scale: 1 (not accurate), 2 (somewhat accurate), and 3 (accurate).

The AAD’s sunscreen FAQs and melanoma FAQs had readability levels of 9.2 and 9.4 (both 9th grade), respectively, and the original ChatGPT sunscreen and melanoma output readability levels were 9.6 and 10.4 (9th grade and 10th grade), respectively, with no differences in readability between AAD and ChatGPT for both question sets (*P*=.32 and *P*=.15, respectively). The first and second prompting of the sunscreen FAQs output generated material at lower reading levels than AAD-generated material (6.0, *P*=.005; 4.4, *P*<.001, respectively). Melanoma FAQs, after prompting, achieved lower reading levels versus AAD material, with scores of 8.0 (8th grade; *P*=.08) and 7.4 (7th grade; *P*=.007) (see Table 1).

The AAD material scored an average of 2.82 in accuracy, while the original ChatGPT material scored 2.89. All of the material (42/42, 100%) averaged within the 2-3 range. Initial and secondary prompting resulted in generated material with average scores of 2.63 and 2.62, respectively. Of the 42 materials generated from prompting, 42 (95.2%) averaged within the 2-3 range.

## Discussion

The AAD’s sunscreen FAQs and melanoma FAQs had readability scores below the recommended threshold of 80 (Flesch Reading Ease scale) and above the recommended 6th-grade reading level, consistent with a study showing that 27 subungual melanoma websites had poor readability overall, with only 22% having readability lower than the 7th-grade reading level [8]. Taken together, these findings emphasize the need to enhance readability of dermatology public education information.

Our study demonstrated that ChatGPT may be a solution to this problem. Prompting ChatGPT following initial inputs improved health information readability versus AAD materials and was closer to or within recommended guidelines. Our findings are similar to a 2023 study assessing 9 uveitis web pages with an average Flesch-Kincaid Grade Level of 11.0 (SD 1.4); ChatGPT improved the readability, with a mean Flesch-Kincaid Grade Level of 8.0 (SD 1.0) [9]. Therefore, the use of ChatGPT to adapt output to enhance readability might have applicability in dermatology and other medical fields.

Most of the ChatGPT-generated material was rated as accurate to somewhat accurate. However, additional prompting resulted in a slight trend toward less accuracy, with 2 responses below the 2-3 (accurate to somewhat accurate) range. This observation may highlight a potential limitation to the applicability of ChatGPT in this context. Additionally, only a small number of questions were assessed. We analyzed the ChatGPT-3.5 version, which includes information up until September 2021.

In conclusion, ChatGPT could be used to enhance the readability of dermatology health information and lower it to the 6th-grade reading level recommended by the AMA. Larger studies are needed to corroborate our data and evaluate the utility of ChatGPT for dermatology public education materials.

## Abbreviations

AAD: American Academy of Dermatology Association
AMA: American Medical Association
